# The benefit of everolimus in recurrent/epithelioid angiosarcoma patients: Case reports and literature review

**DOI:** 10.18632/oncotarget.21832

**Published:** 2017-10-15

**Authors:** Shi-Long Zhang, Li Liang, Yuan Ji, Zhi-Ming Wang, Yu-Hong Zhou

**Affiliations:** ^1^ Department of Hematology, Zhongshan Hospital, Fudan University, Shanghai, China; ^2^ Department of Medical Oncology, Zhongshan Hospital, Fudan University, Shanghai, China; ^3^ Department of Pathology, Zhongshan Hospital, Fudan University, Shanghai, China

**Keywords:** recurrence/metastatic epithelioid angiosarcoma, everolimus, targeted therapy

## Abstract

Epithelioid angiosarcoma (EA) is a kind of rare malignant soft tissue sarcoma, with high recurrence/metastatic rate and poor prognosis. To date, no effective standardized treatment regimen has been available for patients with recurrence/metastatic EA. Everolimus is an oral rapamycin derivative that highly inhibits the mechanistic target of rapamycin(mTOR) signal pathway. Previous studies have suggested that everolimus is effective and safe in some soft tissue sarcoma. We reported two cases with recurrence/metastatic EA, who received everolimus after failure of surgery, radiotherapy, chemotherapy or interventional therapy. Two cases obtained clinical benefit within 1 week, and were evaluated as partial response (PR). The progression free survival (PFS) time was nearly 12.0 and 6.0 months, respectively. The overall survival (OS) time was 18.0 and 10.0 months, respectively. The main adverse event was stomatitis syndrome (grade 1-2), which was well controllable and tolerable. It indicated that everolimus may be more beneficial for recurrence/metastatic EA patients.

## INTRODUCTION

Epithelioid angiosarcoma (EA), which accounts for less than 1% of all soft tissue sarcomas, is an extremely rare malignant tumor derived from vascular endothelial cells or lymphatic endothelial and the literature revealed no clear guidelines as how to treat the disease [1, 2]. EA can occur at any age and affect any organ system, almost 60% of which are cutaneous and commonly found in the face and scalp region [3]. It has high malignant and strong invasiveness, with a high tendency for both local recurrence and distant metastasis and the prognosis is very poor.

At present, the main treatment for EA is surgery and postoperative radiotherapy and (or) chemotherapy in clinical, but as the high invasiveness of the tumor cells, the recurrence rate is very high, most of EA, in particular high-grade tumor, have also associated with poor prognosis and short life expectance [4-6].

In recent years, a more in-depth understanding of the molecular pathophysiology in soft tissue sarcomas has led to increasing exploitation of molecule-targeted novel agents including the mechanistic target of rapamycin (mTOR) kinase inhibitors [7]. Everolimus is the first generation of mTOR inhibitors, and it is a serine-threonine protein kinase in the mTOR signal pathway, which is an important potential target for cancer therapy [8, 9].Some clinical trials have demonstrated the survival benefit of everolimus in soft tissue sarcomas patients. Herein, we report our experience with two patients with recurrent/metastatic EA who achieved long-term disease control with oral everolimus.

## CASE PRESENTATION

**Figure 1:**
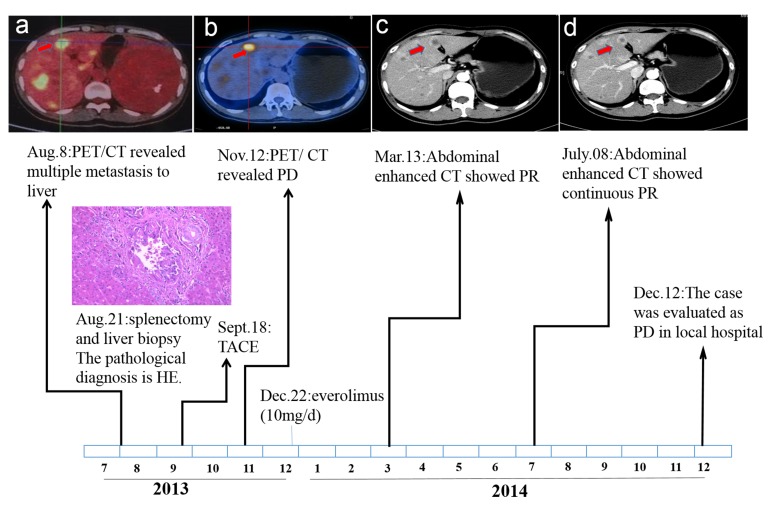
Time sequence scheme of Patient I

**Figure 2:**
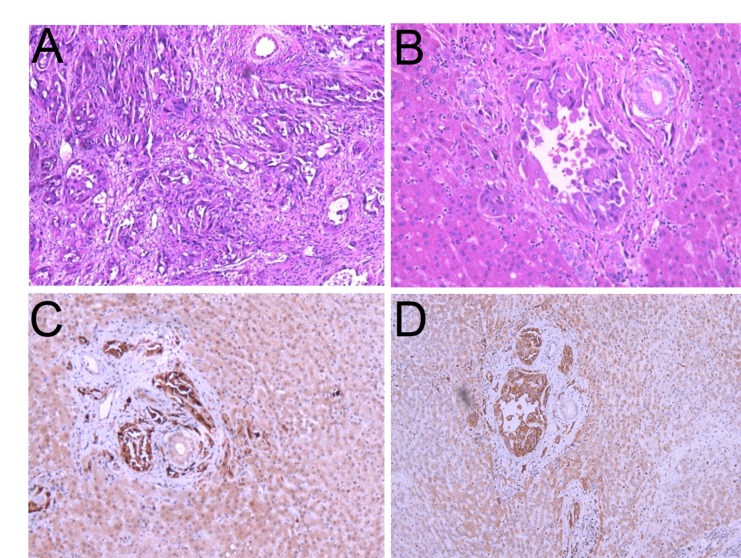
Histologic features of the epithelioid angiosarcoma **A**. and **B**. Hematoxylin-eosin staining of the spleen (A) and liver metastasis (B). **C**. and **D**. Immunohistochemistry (IHC) staining of the primary tumor with different antibodies.4E-BP1 (C) and p70S6K (D).

Patient I, male, 37-year-old, was referred to local hospital on August 2013, due to abdominal pain and liver lesions detected by ultrasonography. Positron emission tomography computed tomography (PET/CT) scan (August 8, 2013) revealed massive occupying lesions of the spleen with increased uptake of fluorodeoxyglucose (FDG) and multiple metastasis to liver (Figure 1a). Subsequently, he underwent splenectomy along with liver biopsy (August 21, 2013). The pathological result was primary EA in spleen (Figure 2A) and multiple metastasis to liver (Figure 2B). The immunohistochemistry (IHC) staining revealed positive expression of the phosphorylation of eukaryotic translation initiation factor 4E (eIF4E)-binding protein (4E-BP1) (Figure 2C), p70S6 kinase (p70S6K) (Figure 2D), D2-40, CD31 and CD34, and negative expression of smooth muscle actin (SMA), human melanoma black 45 (HMB 45), S100 and Desmin. On September 18,2013, The patient received transhepatic arterial chemotherapy and embolization (TACE) with no further treatment. After 3 months (November 12, 2013), PET/CT exhibited that multiple hepatic malignancies with the longest diameter of 3.2cm (Figure 1b). He was evaluated as disease progression (PD). The patient visted to our department on December 19, 2013. Comprehensive physical examination showed no positive sign, and Eastern Cooperative Oncology Group (ECOG) performance status was 1 point. The pain mainly occurred in right upper abdomen with a score of 3-4 points by digital hierarchy process (NRS). The patient started oral administration of everolimus (10mg/d) on December 22, 2013. After 3 months, CT scan (March 13, 2014) showed the lesions were obviously shrink with the longest diameter of 1.5 cm (Figure 1c). And the case was assessed as partial response (PR). Seven months later, the CT scan (July 08, 2014) showed the lesions were similar to latest images (Figure 1d), and the case was evaluated as continuous PR. During the medication, no obvious adverse event was observed except for stomatitis syndrome (grade 1). Moreover, the patient had no chief complaints and was in good physical condition with 0 point of ECOG. the case had achieved a progression free survival (PFS) of close to 12.0 months, before PD on December 12, 2014 (Figure 1). The overall survival (OS) time was 18.0 months in the end.

**Figure 3:**
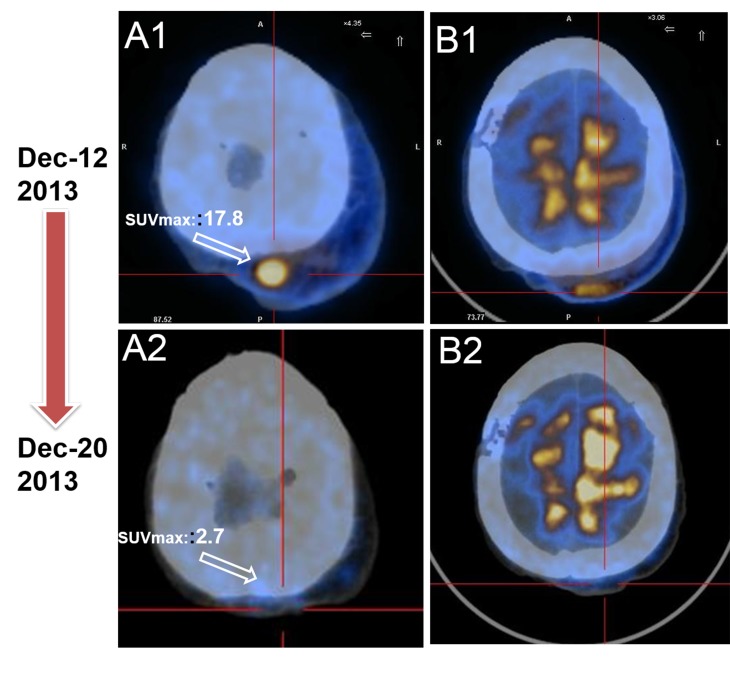
PET/CT serves as a favorable tool to evaluate the early therapy effect for EA patient **A**. Baseline PET/CT image before everolimust treatment revealed masses on left upper scalp, and the SUVmax was 17.8 ( December 8, 2013). **B**. PET/CT displayed significant decreased tumor burden with everolimus,and the SUVmax was 2.7.The case was evaluated as PR (Dec 20, 2013).

Patient II, female, 61-year-old, was examined by scalp biopsy (November 2012) in local hospital due to multiple scalp and facial masses for more than 6 months. Pathological report concluded EA in the scalp region. The IHC staining showed positive expression of 4E-BP1, Vimetin, CD31 and CD34, and negative expression of Syn, CD30, SMA, HMB 45, S100 and Desmin. The patient underwent expanded excision of the tumors followed by radiotherapy combined with chemotherapy of paclitaxel for 2 cycles (January 2013 to February 2013). Six months after the final administration of chemotherapy, recurrence disease was evaluated because multiple masses were found in the scalp. She was admitted to our department on December 8, 2013. Comprehensive physical examination showed no positive sign, and ECOG performance status was 2 points. PET/CT (December 10, 2013) showed soft tissue masses in the left upper scalp with the longest diameter of 13.5cm. And the maximum standardized uptake value (SUVmax) was about 17.8 (Figure 3A1 and 3B1). From December 12, 2013, she took everolimus orally (10 mg/d). The soft tissues masses reduced slightly in 3 days. Just 10 days later, PET/CT (December 20, 2013) indicated significant tumor shrinkage with slightly increased uptake of FDG. The SUVmax was about 2.7 (Figure 3A2 and 3B2). And the therapeutic evaluation was PR. After half a month, the dose was reduced to 5 mg/d due to stomatitis syndrome (grade 2) that cause eating disorder. And six months later, CT indicated that disease was PD. Accordingly, The PFS of the patient was 6.0 months after taking everolimus. The OS was 10.0 months in the end.

## DISSCUSSION

EA is a rare and high-invasive tumor that some cases occur earlier distant metastasis, with poor prognosis [10]. The common treatment for EA mainly contains traditional surgery resection, radiotherapy and chemotherapy-based comprehensive treatment. However, most of them usually result in serious poisonous events and high recurrence, highlighting the need to develop new strategies [11, 12]. According to the results of the current clinical studies, targeted therapy has satisfactory effect on soft tissue sarcomas patients, such as the mTOR inhibitor everolimus.

The mTOR is a serine-threonine protein kinase that acts as a central regulator in a variety of processes including gene transcription, protein synthesis, cell apoptosis and some other vital processes [13]. Aberrant transduction and regulation of mTOR signal pathway keep close with the occurrence and progression of many cancers [14]. It is considered that inhibition of mTOR pathway leads to an effective block of abnormal signal transduction and prevent the progression of tumor cells [15, 16].Therefore, the inhibition of this pathway has been regarded as one of the most promising therapeutic strategies for advanced sarcomas [17].

**Figure 4:**
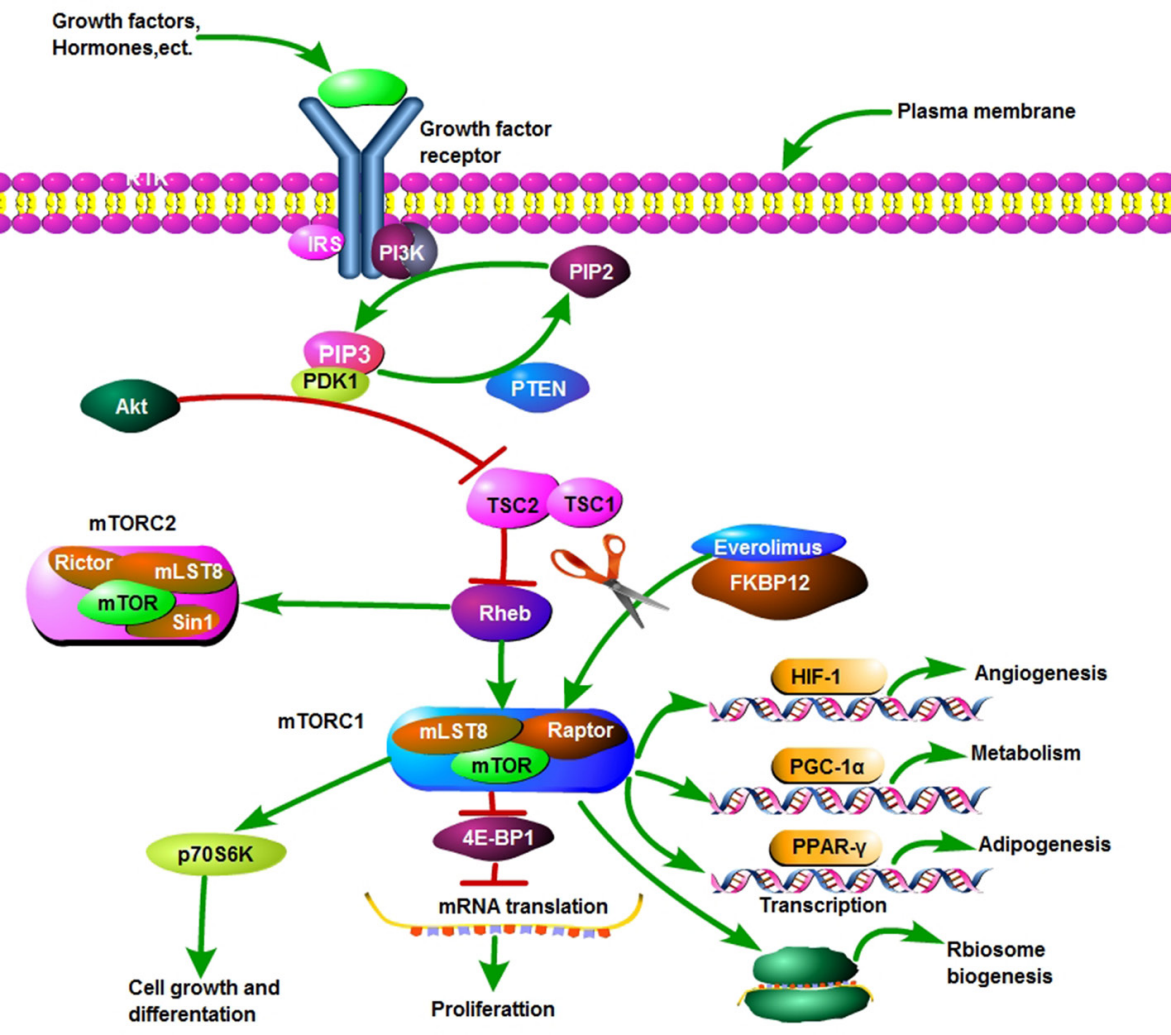
The PI3k-Akt-mTOR pathway

Everolimus is (RAD001; Novartis Pharmaceuticals) is an oral rapamycin derivative that leads to anticancer actions by forming mTOR complex 1 (mTORC1) with FK506 Binding Protein 12 KD (FKBP12). In addition to immunosuppression [18], everolimus shows an ability of continuously inhibiting mTOR via the phosphatidylinositol-3kinase (PI3K)-Akt-mTOR signal pathway, which may contribute to its triple role of inhibiting cell growth and proliferation, nutrition metabolism and angiogenesis of tumors [19] (Figure 4). In this report, patient I obtained a good clinical efficacy when 4E-BP1, p70S6K showed positive. For patient II, everolimus shrink the tumor with lower FDG in three days, which may directly result from inhibiting mTOR signal pathway. To date, everolimus has been approved by the US Food and Drug Administration (FDA) to treat advanced renal cell carcinoma after progression on sunitinib or sorafenib, pancreatic neuroendocrine tumors, and aromatase inhibitor (AIs)-refractory HR+/HER2- breast cancer [20-22].

Several clinical trials have been conducted to evaluate the effect and safety of everolimus for patients with advanced solid tumors. A phase I studies was conducted for patients with advanced solid tumors (eg, colorectal, non-small cell lung, and breast) who were unresponsive to standard therapy and demonstrated that everolimus is dose-dependent and the optimal schedule was 10 mg once daily. The adverse events are dose-limiting and contain stomatitis syndrome, thrombocytopenia, gastritis and hemorrhagic gastritis [23]. In patients with advanced, progressive neuroendocrine tumors, it was also confirmed that median PFS was significantly improved in the everolimus group when compared with the placebo group (11.0 months *vs*. 3.9 months, *p* <0.001) [24].

To further explore the indications of everolimus, a multicenter phase II trial for 41 heavily pretreated patients with bone and soft-tissue sarcoma was conducted. In this trial, everolimus was given at 10mg/d and showed good anti-tumor effect with well-controlled toxicities. The PFS at 16 weeks was 27%, and was highest in EA patients (2/3). With a median follow-up of 10.9 months, the median PFS and OS was approximately 2.0 months and 6.0 months, respectively. The grade 3/4 adverse events contained hyperglycemia in 15% of patients, stomatitis syndrome 7%, pain about 5% and asthenia 5%, most of which were mild and manageable [25]. Recently, everolimus has been investigated in varieties of recurrent/metastatic tumors, including recurrent ovarian clear cell carcinoma [26], relapsed or refractory chronic lymphocytic leukemia [27] and hepatocellular carcinoma [28], and brought new hope for cancer.

To our best knowledge, few large clinical studies has been conducted to investigate the effect and safety of everolimus on EA patients especially for those who had underwent recurrence/metastasis after failure of surgery, radiotherapy, chemotherapy or interventional therapy. This is the first case report of recurrent/metastasis EA patients treated with everolimus. Here we present our experience. The reasons for everolimus used in the 2 cases were based on previous studies of everolimus in soft-tissue sarcoma and unsatisfactory outcome with previous treatment. Given the general condition of patients and the attitudes of the patient and family members, we treated them with everolimus in a daily dose of 10 mg. After administration for some time, the disease was well controlled as PR and the PFS of the patients were nearly 12.0 and 6.0 months, respectively. In particular, both patients had pain relief and improved quality of life rapidly in three days after oral everolimus. Just 10 days later, the PET/CT imaging of patient II displayed shrunk tumors and The SUVmax greatly reduced from 17.8 to 2.7. It indicated that PET/CT is a sensitive and early predictor of the therapy response for EA patient, albeit at heavy cost in the developing world. Although the 2 cases reported in this study were individual, everolimus did display its satisfactory efficacy and safety in recurrent/metastasis EA. However, Further studies are necessary to establish the definite role of everolimus in recurrent/metastasis EA patients.

The common adverse events of everolimus were hyperglycemia, stomatitis syndrome and asthenia, yet most of them were controllable and tolerable. And it was suggested that a dose of 5-10 mg/d was of comparatively safety and well tolerance [23]. In our cases, stomatitis syndrome with grade 1-2 happened, and it got well management. Remarkably, no significant difference was detected in fasting blood glucose, total cholesterol and triglyceride during treatment.

In recent years, the combination of chemotherapy and molecular targeted therapy in soft tissue sarcoma has always been a research focus. In the light of the good results of everolimus on other cancers, we can put forward two questions boldly: (1) Inspired by the satisfactory outcomes of everolimus in the BOLERO-2 trial [29], whether the combination of everolimus and current standard chemotherapy can improve the survival benefits in patients with recurrence/metastatic EA? (2) Possible prognostic biomarkers including PTEN deletion, PIK3CA mutations, p70S6K and 4E-BP1 for tumor response to mTOR inhibitors have been illustrated in glioblastoma, breast and prostate cancer cells cultured in vitro [30, 31], whether these biomarkers may contribute to the prognosis and individual therapy of EA in clinic? . In order to answer the questions, more high-quality basic and clinical studies are warranted to carry out and these questions may well further provide clinical guidelines in the future.
